# Independent associations and effect modification between lifetime substance use and recent mood disorder diagnosis with household food insecurity

**DOI:** 10.1371/journal.pone.0191072

**Published:** 2018-01-23

**Authors:** Karen M. Davison, Cliff Holloway, Lovedeep Gondara, Anne S. Hatcher

**Affiliations:** 1 School of Nursing, University of British Columbia, Vancouver, British Columbia, Canada; 2 Health Science Program, Department of Biology, Kwantlen Polytechnic University, Surrey, British Columbia, Canada; 3 Fulbright Canada Research Chair, University of Hawai`i at Mānoa, Honolulu, Hawai`i, United States of America; 4 Faculty of Health Sciences, University of Ontario Institute of Technology, Oshawa, Ontario, Canada; 5 School of Computing Science, Simon Fraser University, Burnaby, British Columbia, Canada; 6 Department of Human Services, Center for Addiction Studies, Metropolitan State University Denver, Denver, Colorado, United States of America; Chiba Daigaku, JAPAN

## Abstract

Poor mental health and substance use are associated with food insecurity, however, their potential combined effects have not been studied. This study explored independent associations and effect modification between lifetime substance use and mood disorder in relation to food insecurity. Poisson regression analysis of data from British Columbia respondents (n = 13,450; 12 years+) in the 2007/08 Canadian Community Health Survey was conducted. Measures included The Household Food Security Survey Module (7.3% food insecure), recent diagnosis of a mood disorder (self-reported; 9.5%), lifetime use of cannabis, cocaine/crack, ecstasy, hallucinogens, and speed, any lifetime substance use, sociodemographic covariates, and the interaction terms of mood disorder by substance. For those with recent diagnosis of a mood disorder the prevalence of lifetime substance use ranged between 1.2 to 5.7% and were significantly higher than those without recent mood disorder diagnosis or lifetime use of substances (p’s < 0.05). For respondents with a recent mood disorder diagnosis or who used cannabis, food insecurity prevalence was higher compared to the general sample (p < 0.001); prevalence was lower for cocaine/crack use (p < 0.05). Significant effect modification was found between mood disorder with cannabis, ecstasy, hallucinogen and any substance use over the lifetime (PRs 0.51 to 0.64, p’s 0.022 to 0.001). Independent associations were found for cocaine/crack and speed use (PRs 1.68, p’s < 0.001) and mood disorder (PRs 2.02, p’s < 0.001). Based on these findings and the existing literature, future study about coping and resilience in the context of substance use, mental health, and food insecurity may lead to the development of relevant interventions aimed at mental well-being and food security.

## Introduction

Food insecurity–which occurs when people are physically or economically unable to consume a diet of sufficient quantity of food, or have uncertainty in their ability to do so [1]–profoundly impacts mental well-being. Several studies have reported associations between food insecurity and various indicators of poor mental health such as depression, suicide ideation and substance use and some of these relationships may be bidirectional [2–7]. A better understanding of these associations could help provide the information needed to develop comprehensive policy and program interventions that would mitigate food insecurity.

**Fig 1:**
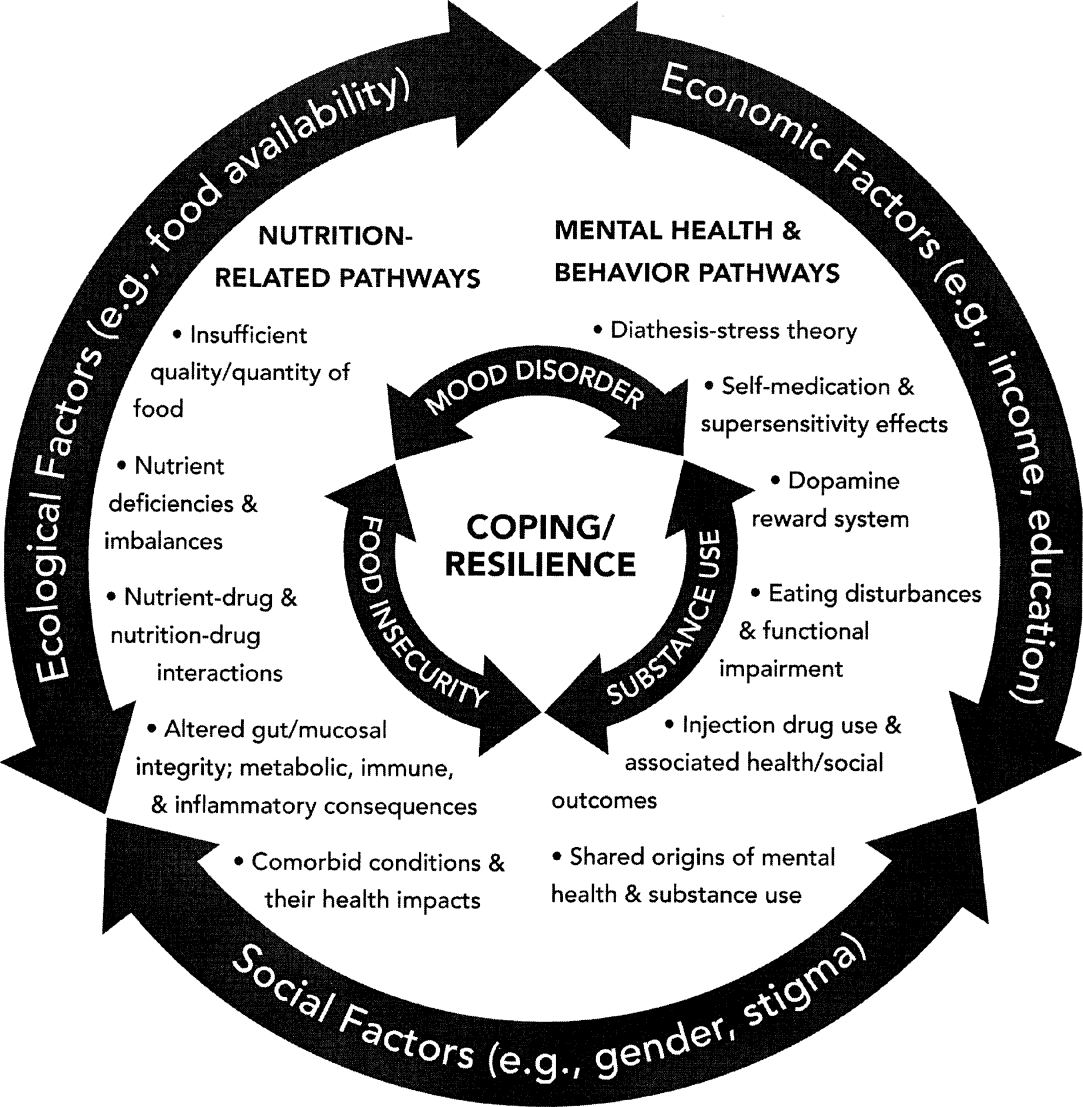
Food insecurity, poor mental health, and substance use: Explanatory pathways [9, 45].

While studies about food insecurity and mental health have often controlled for substance use as a potential confounder, no studies have measured for possible effect modification between poor mental health and substance use. A number of mechanisms related to economic (income, education), social (gender, stigma), macro-level drivers (e.g., food environments), and biological pathways (e.g., stress response, inadequate nutrients) [8, 9] explain potential inter-relations among poor substance use, mental health, and food insecurity (Fig 1). Poor mental health among those who are food insecure [10–12] can contribute to high stress levels, challenges in coping, and increased risk to use substances. Studies about food insecurity and injection drug use suggest that between 30% and 70% of individuals who use drugs report some level of food insecurity [2, 13–18]. Nutritional inadequacy, which is often correlated with food insecurity [19] and substance use [20–24], is linked with poor mental health [25–28]. Stress, due to feelings of deprivation and/or anxiety about food supply [7, 29–31], can be a contributor to or outcome of food insecurity, mental health, and/or substance use.

Mental health symptoms (e.g., loss of interest in usual activities, impairment in cognition) [10] can also alter food skills (e.g., reduced concentration to follow recipes) and choices (e.g., selection of more convenient options), reduce income, and/or impact housing and accommodation [32] and thereby contribute to food insecurity. Structural based stresses, including insufficient income, poor housing, lack of secure, well-paid employment, and inadequate social support further compound challenges related to food insecurity, coping, and mental health [33].

It is well established that poor mental health and substance use frequently co-occur [34–36]. Substance use may be a cause or consequence of mental ill health or share a common origin with mental ill health. An alternative explanation is that mental ill health and substance use interact and maintain each other [37]. Given these reported relationships, it would seem reasonable to expect that substance use would be a direct effect modifier of mental health in relation to the outcome of food insecurity. Applying an effect modification analysis to examine the associations between substance use and mental health could help to address the complex issue of food insecurity in and lead to the development of effective prevention tools in vulnerable subpopulations.To better understand these relationships, national data that provides measures of previous substance use, recent mood disorder diagnosis (past 12 months), and household food security (past 12 months) was analyzed to examine: 1) the prevalence of food insecurity among those with recent diagnosis of a mood disorder and that reported lifetime use of selected substances; 2) effect modification between lifetime substance use and recent mood disorder diagnosis in relation food insecurity. In this analysis prior substance use is considered a direct effect modifier of mental health (exposure variable) and its relationship to food insecurity (outcome). We hypothesized that: 1) the prevalence of food insecurity would be higher in those with a recent diagnosis of a mood disorder and that reported lifetime substance use compared to those without a recent diagnosis of a mood disorder or reported lifetime substance use; and 2) there would be significant effect modification between all types of substance use and recent mood disorder diagnosis in relation to food insecurity.

## Materials and methods

### Sampling method and sample

The sample (Canadians 12 years+) was derived from the 2007/08 (Cycles 4.1/4.2) Canadian Community Health Survey (CCHS). Respondents are selected using a multi-stage sampling strategy [38] and do not include persons living on reserves and other Aboriginal settlements in the provinces; full-time members of the Canadian Forces; the institutionalized population; and children aged 12–17 that are living in foster care. Collectively, these exclusions represent less than 3% of the Canadian population aged 12 and over. At the time of household contact for data collection, the interviewer sees a list of all members of the household listed and a person aged 12 years or over is automatically selected using various selection probabilities based on age and household composition. All Individuals sampled, including those between 12 to 18 years, answered the questions on their own. However, for some questions (e.g., income) consulation with a parent or guardian may have been necessary. The study sample were respondents from British Columbia, a province which opted to include measures of substance use (n = 13,450). Approval for use of the de-identified CCHS dataset was granted by Statistics Canada. All data was vetted by a Statistics Canada analyst prior to release to ensure that respondent privacy was maintained.

### Measurements

#### Food insecurity

Food insecurity was measured using the 18-item Household Food Security Survey Module (HFSSM) [39]. To help reduce respondent burden, CCHS respondents were first screened based on responses to either of the "food sufficiency questions" which asked whether the household, in the past 12 months sometimes did not have enough to eat or often did not have enough to eat. Those who answered affirmatively to the screening questions were then administered the HFSSM. The food security classifications used for this study included: 1) Food secure: 0 or 1 affirmative response on HFSSM’s adult and/or child scale; indicates access, at all times in the previous year, to enough food for an active, healthy life for all household members; 2) Food insecure: included categories of moderate (2 to 5 affirmative responses on HFSSM’s adult and/or child scale) or severe food insecurity (> 6 affirmative responses on HFSSM’s adult and/or child scale); indicates that any household member had compromised in quality and/or quantity of food consumed which may have disrupted eating patterns.

#### Mood disorder

A composite variable to describe presence of a mood disorder was based on responses to two survey questions: 1) a question that asked whether an individual had been diagnosed with a mood disorder such as depression, bipolar disorder, mania, or dysthymia by a healthcare professional in the past 12 months; plus 2) a question that asked about having an anxiety disorder such as a phobia, obsessive- compulsive disorder or a panic disorder. We compared results of analysis (substance use in relation to food insecurity) between those diagnosed with mood disorder versus those with a diagnosed mood disorder that also indicated they had other mental health conditions and found no significant differences. Therefore respondents who indicated they had been diagnosed with a mood disorder in the past 12 months, including those with reported comorbidities of anxiety, obsessive-compulsive or panic disorder, were included in the analysis.

#### Substance use

Assessment of substance use was based on questions derived from Canada’s Alcohol and Other Drugs Survey [40] that asked about lifetime use of: 1) marijuana, cannabis or hashish (> once); 2) cocaine or crack; 3) speed (amphetamine); 4) ecstasy (3,4-methylenedioxy-methamphetamine) or similar drugs; or 5) hallucinogens, phencyclidine (PCP) or lysergic acid diethylamide (LSD). We analyzed each substance separately as current literature is limited about their relationships with food insecurity. We also analyzed a composite variable that measured any lifetime substance use; a derived variable from the survey. While data on other substances (e.g., steroids) was collected and the opportunity existed to also examne polysubstance use, the frequencies on these measures were too low to enable analysis.

#### Covariates

Covariates were selected based on a literature search of factors that may confound or mediate the relationships among food insecurity, mental health, and substance use. The covariates sex, age (categorized as < 18 years, 18 to 29 years, 30 to 39 years, 40 to 49 years, 50 to 59 years, and > 60 years), education (four categories: no post- secondary degree, certificate or diploma; secondary school graduation, no post- secondary education; some post-secondary education; or post-secondary degree/diploma), and relationship status (in a relationship as married or common-law versus not in a relationship that included widowed, separated, divorced, single, never married). We also included the covariate income, dichotomized as low versus adequate income, that was based on a derived variable which measured the ratio between the total income of the respondent's household and the low income cut-off corresponding to the number of persons in the household and the size of the community.

### Data analysis

The secured data was analyzed using STATA 11.0 [41] in a Statistics Canada Research Data Center. The bootstrap method was used to account for the complex survey design [42]. Descriptive analysis was done for all variables. Bivariate and stratified analyses were done to assess relationships between all variables and food insecurity. Using Poisson regression with robust variance, adjusted prevalence ratios (proportion with food insecurity/proportion exposed to a substance during their lifetime and proportion with food insecurity/proportion diagnosed with a mood disorder in the previous year) were analyzed. Poisson regression was selected for this cross-sectional data as the exposure (e.g., lifetime substance use) refers to the beginning of the presumed individual risk periods.

In total, six models were analyzed; one for each of the five substances and one which included a composite variable of any lifetime substance use. In each model, interaction terms were generated for substance by mood disorder in order to test for effect modification. The analysis for each of the six models first consisted of including all individual variables and the interaction terms (full model). If the interaction terms were not significant, then subsequent analyses were conducted with the non-significant interaction terms removed. Goodness-of-fit chi-squared tests assessed for model fit.

## Results

In the sample of 13,450 individuals from British Columbia, 7.3% (95% CI 6.9–7.7) were food insecure. Of those who were recently diagnosed with a mood disorder (n = 1280), the prevalence of food insecurity was significantly higher compared to the general sample (19.0%, 95% 16.8–21.1, p < 0.0001). In addition, those with recent mood disorder diagnosis had significantly higher prevalence estimates of lifetime use for each substance (1.2 to 5.7%) compared to those without recent mood disorder diagnosis or lifetime use of substances (p’s < 0.05). Of those who reported having a mood disorder, there were significant differences according to age, relationship status, income level and food insecurity status (p’s < 0.05) (Table 1) compared to those without a mood disorder. Of the different types of substances used by those who reported having a mood disorder, the prevalence of food insecurity (13.7%, 95% CI 11.8–15.6) was significantly higher for cannabis use versus non-use (5.9%, 95% CI 4.6–7.2, p < 0.001). Conversely, the prevalence of food insecurity (6.9%, 95% CI 5.5–8.3) was significantly lower for cocaine and crack use versus non-use (12.7%, 95% CI 10.9–14.6, p < 0.05).

**Table 1:** Sample and substance use characteristics by food security status in individuals diagnosed with a mood disorder in past 12 months^a^.

	Diagnosis of mood disorder in past 12 months		
Characteristic	Food secure	Food insecure	^χ2^, p-value
Sex			0.90, p = 0.342
Males	28.5	5.9	
Females	52.6	13.1	
Age			2.76, p = 0.021
12 to 18 years	3.9	1.0	
18 to 29 years	1.1	4.2	
30 to 39 years	14.8	3.6	
40 to 49 years	18.5	4.9	
50 to 59 years	15.4	3.6	
> 60 years	17.3	1.8	
Education			0.94, p = 0.423
No post-secondary degree, certificate or diploma	14.1	3.9	
Secondary school graduation, no post-secondary education	13.7	4.0	
Some post-secondary education	9.9	2.4	
Post-secondary degree/diploma	43.2	8.8	
Relationship Status			11.86, p < 0.001
In relationship (married, or common-law)	47.3	6.1	
Not in a relationship (widowed, separated, divorced, single, never married)	33.7	13.0	
Income			92.50, p < 0.001
Adequate income	70.8	9.4	
Low income	10.2	9.6	
Substances: Lifetime Use			12.46, p = 0.001
Marijuana, cannabis or hashish (more than once)			
Yes	42.6	13.7	
No	37.9	5.9	
Cocaine or crack			8.68, p = 0.003
Yes	16.9	6.9	
No	63.6	12.7	
Speed (amphetamines)			3.59, p = 0.059
Yes	9.0	3.4	
No	71.7	16.9	
Ecstasy (3,4-methylenedioxy-methamphetamine; MDMA) or other similar drugs			1.75, p = 0.186
Yes	10.5	3.6	
No	70.0	15.9	
Hallucinogens, PCP (phencyclidine), or LSD (Lysergic acid diethylamide; acid)			2.37, p = 0.125
Yes	16.6	5.2	
No	63.9	14.3	
Lifetime use of any substance			9.00, p = 0.003
Yes	46.0	13.9	
No	34.5	5.7	

^a^Totals for some variables may not exactly be 100 due to rounding

Based on the Poisson regression analysis (Table 2), significant independent associations were found between cocaine/crack (PR 1.68, 95% CI 1.33–2.12), p < 0.001) and speed (PR 1.67, 95% CI 1.29–2.17, p < 0.001) in relation to food insecurity. In the model including cocaine/crack, recent mood disorder diagnosis (PR 2.02, 95% CI 1.64–2.49, p < 0.001), age (PR 0.81, 95% CI 0.77–0.85, p < 0.001), relationship status (PR 1.53, 95% CI 1.22–1.92, p < 0.001), education (PR 1.62, 95% CI 1.30–2.01, p < 0.001), and income (PR 4.29, 95% CI 3.53–5.22, p < 0.001) were also significantly associated with food security status. Similarly, in the model including speed, recent mood disorder diagnosis (PR 2.13, 95% CI 1.75–2.59, p < 0.001), age (PR 0.81, 95% CI 0.77–0.85, p < 0.001), relationship status (PR 1.54, 95% CI 1,23–1.93, p < 0.001), education (PR 1.63, 95% CI 1.30–2.04, p < 0.001), and income (PR 4.29, 3.50–5.21, p < 0.001) were also significantly associated with food security status. Significant effect modification was indicated between presence of mood disorder with cannabis use (0.64, 95% CI 0.43–0.94, p = 0.022), hallucinogen use (0.64, 95% CI 0.41–0.98, p = 0.041), ecstasy use (0.57, 95% CI 0.33–0.97, p = 0.037), and any lifetime substance use (0.51, 95% CI 0.35–0.76, p<0.001) in relation to food insecurity. The goodness-of-fit chi-squared test results were not significant for all final models suggesting that the Poisson model forms fit the data.

**Table 2:** Poisson regression results for food insecurity, mood disorder, and substance—full and reduced models.

Variable (baseline)	Full Model		Reduced Model	
	Prevalence Ratio (95% CI)	p-value	Prevalence Ratio (95% CI)	p-value
Marijuana, cannabis or hashish (more than once)				
Mood disorder (not diagnosed in previous 12 months)	2.68 (1.93–3.70)	<0.001	2.59 (1.89–3.55)	<0.001
Substance (no lifetime use)	2.42 (1.57–3.75)	<0.001	1.95 (1.59–2.40)	<0.001
Sex (male)	1.16 (0.89–1.52)	0.277	0.92 (0.77–1.12)	0.414
Age (12 to 18 years)	0.82 (0.77–0.88)	<0.001	0.82 (0.78–0.87)	<0.001
Relationship (single)	1.58 (1.26–1.97)	<0.001	1.55 (1.24–1.95)	<0.001
Education (no high school)	1.74 (1.39–2.18)	<0.001	1.71 (1.37–2.14)	<0.001
Income (low)	4.87 (3.50–6.79)	<0.001	4.41 (3.61–5.39)	<0.001
Substance x mood disorder	0.61 (0.40–0.92)	0.019	0.64 (0.43–0.94)	0.022
Cocaine or crack				
Mood disorder (not diagnosed in previous 12 months)	2.33 (1.82–2.99)	<0.001	2.02 (1.64–2.49)	<0.001
Substance (no lifetime use)	2.29 (1.30–4.01)	0.004	1.68 (1.33–2.12)	<0.001
Sex (male)	0.99 (0.80–1.22)	0.930	0.94 (0.78–1.13)	0.512
Age (12 to 18 years)	0.81 (0.77–0.86)	<0.001	0.81 (0.77–0.85)	<0.001
Relationship (single)	1.53 (1.22–1.92)	<0.001	1.53 (1.22–1.92)	<0.001
Education (no high school)	1.64 (1.32–2.05)	<0.001	1.62 (1.30–2.01)	<0.001
Income (low)	4.56 (3.59–5.80)	<0.001	4.29 (3.53–5.22)	<0.001
Substance x mood disorder	0.67 (0.43–1.06)	0.084	—	—
Speed (amphetamines)				
Mood disorder (not diagnosed in previous 12 months)	2.23 (1.79–2.78)	<0.001	2.13 (1.75–2.59)	<0.001
Substance (no lifetime use)	2.48 (1.14–5.37)	0.022	1.67 (1.29–2.17)	<0.001
Sex (male)	0.97 (0.79–1.18)	0.748	0.96 (0.80–1.15)	0.635
Age (12 to 18 years)	0.81 (0.77–0.85)	<0.001	0.81 (0.77–0.85)	<0.001
Relationship (single)	1.54 (1.23–1.92)	<0.001	1.54 (1.23–1.93)	<0.001
Education (no high school)	1.64 (1.31–2.06)	<0.001	1.63 (1.30–2.04)	<0.001
Income (low)	4.40 (3.56–5.45)	<0.001	4.27 (3.50–5.21)	<0.001
Substance x mood disorder	0.77 (0.44–1.36)	0.371	—	—
Ecstasy (3,4-methylenedioxy-methamphetamine; MDMA) or other similar drugs				
Mood disorder (not diagnosed in previous 12 months)	2.43 (1.98–2.99)	<0.001	2.43 (1.97–2.98)	<0.001
Substance (no lifetime use)	2.12 (1.23–3.64)	0.007	2.83 (1.99–4.02)	<0.001
Sex (male)	0.95 (0.78–1.16)	0.617	0.96 (0.80–1.15)	0.679
Age (12 to 18 years)	0.82 (0.78–0.87)	<0.001	0.83 (0.79–0.87)	<0.001
Relationship (single)	1.51 (1.21–1.89)	<0.001	1.51 (1.21–1.89)	<0.001
Education (no high school)	1.62 (1.30–2.03)	<0.001	1.62 (1.30–2.03)	<0.001
Income (low)	4.84 (3.86–6.07)	<0.001	4.86 (3.87–6.09)	<0.001
Substance x mood disorder	0.53 (0.30–0.93)	0.027	0.57 (0.33–0.97)	0.037
Hallucinogens, PCP (phencyclidine), or LSD (lysergic acid diethylamide; acid)				
Mood disorder (not diagnosed in previous 12 months)	2.37 (1.90–2.96)	<0.001	2.36 (1.90–2.94)	<0.001
Substance (no lifetime use)	3.56 (2.07–6.12)	<0.001	2.40 (1.81–3.16)	<0.001
Sex (male)	0.98 (0.80–1.21)	0.861	0.94 (0.78–1.13)	0.501
Age (12 to 18 years)	0.82 (0.78–0.86)	<0.001	0.81 (0.77–0.85)	<0.001
Relationship (single)	1.57 (1.26–1.97)	<0.001	1.56 (1.25–1.95)	<0.001
Education (no high school)	1.64 (1.32–2.06)	<0.001	1.66 (1.33–2.08)	<0.001
Income (low)	4.66 (3.69–5.88)	<0.001	4.64 (3.68–5.84)	<0.001
Substance x mood disorder	0.64 (0.42–0.99)	0.043	0.64 (0.41–0.98)	0.041
Use of any substance over the lifetime (n = 13445)				
Mood disorder (not diagnosed in previous 12 months)	2.94 (2.11–4.11)	<0.001	2.98 (2.16–4.12)	<0.001
Substance (no lifetime use)	2.63 (1.71–4.05)	<0.001	2.48 (1.91–3.23)	<0.001
Sex (male)	1.18 (0.90–1.55)	0.231	1.18 (0.90–1.55)	0.237
Age (12 to 18 years)	0.83 (0.78–0.88)	<0.001	0.83 (0.79–0.87)	<0.001
Relationship (single)	1.57 (1.26–1.96)	<0.001	1.56 (1.25–1.96)	<0.001
Education (no high school)	1.75 (1.39–2.19)	<0.001	1.76 (1.40–2.20)	<0.001
Income (low)	4.84 (3.45–6.78)	<0.001	4.32 (3.54–5.28)	<0.001
Substance x mood disorder	0.54 (0.35–0.81)	0.003	0.51 (0.35–0.76)	<0.001

## Discussion

Significant effect modification was found between recent mood disorder diagnosis and lifetime use of cannabis, hallucinogens, ecstasy, and any substance use over the lifetime in relationship with food insecurity were found; all of these PRs were less than the null value. Conversely, independent associations were found for crack/cocaine and speed use. Several correlates, such as age, education, and income were also significantly associated with food insecurity. These results help illustrate how effect modification analyses may highlight potential early indicators of vulnerability to food insecurity [43].

Interestingly, the significant findings for effect modification appear to suggest that lifetime use of cannibis, hallucinogens, or ecstasy was protective against recent mood disorder diagnosis and food insecurity. The explanation for this relationship is not readily apparent. Prior studies have not reported results about effect modification of substance use and mental health in which comparisons can be made. It is possible that survey respondents may have used substances infrequently (e.g., experimented) and their effects were non-existent or minimal. Data about frequency and amount of use of the substances and their relationship with mental health and food insecurity would help ascertain the accuracy of this assumption. Other research has suggested effect modification occurs with age when examining associations between mental health and substance use [44] suggesting that future study should focus on effects specific to adolesence, young adulthood, and adulthood.

While this investigation suggests exposure to substance use and/or poor mental health are associated with food insecurity, there are potentially multiple directions of association among these variables. As previously indicated in the introduction, relationships among food insecurity, mental health, and substance use (Fig 1) may be due to independent or synergistic effects of various nutrition, mental health, and behavioural pathways [9, 45]. Structural level drivers that include ecological (e.g., food environments), economic (e.g., income), and social factors (e.g., stigma) may intersect with these pathways [46]. The trajectories and final outcomes depend on one’s coping and resilience [7, 47, 48]. For example, food insecurity can contribute to over nutrition (overweight, obesity), and under nutrition (protein and energy deficiencies), nutrient excesses and disproportions, eating disturbances, micronutrient deficiencies, and alterations in the gut-microbiota-brain axis [49–52] which can compromise nutritional and mental health [4]. Nutritional status may be further undermined due to drug (e.g., substance, psychiatric medication) and nutrition interactions, [29, 53], eating disturbances (e.g., fasting to heighten drug effects) [54, 55], and imbalances in metabolic responses [56, 57]. Conversely, mental health pathways such as diathesis–stress may explain how predispositional vulnerability to poor mental health may be triggered by environmental influences (e.g., stressors such as food insecurity) [58] leading to subsequent substance use. Furthermore, mental ill health may heighten a substance’s effects due to “supersensitivity” [59] and lead to sustained drug use as mental health problems persist with continued substance use and continued substance use alleviate psychiatric symptoms [37]. To explore this investigation’s findings further, relevant literature related to the five substances studied is highlighted.

### Effect modification: Mood disorder and substances

Effect modification between mood disorder and cannabis may be explained by the self medication hypothesis. Cannabinoid effects include euphoria and therefore may be used as a means to control symptoms of a mood disorder. However, experimental studies have shown association between the psychoactive component delta-9-tetrahydrocannabinol, psychosis and schizophrenia-like symptoms [60, 61]. Evidence from prospective studies suggest a causal link between cannabis use and psychosis in some individuals that are genetically vulnerable [62, 63].

Cannabis use stimulates appetite and can increase caloric intake by 40% [64]. This creates greater demands for food intake which can heighten food insecurity. Hallucinogens, such as LSD and PCP, and stimulants such as MDMA can produce substantial anorexia which may change the perception of hunger [65]. In other words, the conditions leading to food insecurity may not have changed, but the altered state brought on by the substance has changed an individual’s perception of this state. The use of hallucinogens may trigger onset of psychosis [37] and worsen mental health [66] through mechanisms such as altered glutamatergic neurotransmission [67] which may perpetuate substance use. Some individuals that use MDMA heavily experience long-lasting depression [68].

### Independent effects: Crack/cocaine or speed (methamphetamine)

Cocaine suppresses appetite and contributes to eating disturbances [37]. ‘Drug binges’–patterns of intense drug use often lasting for days at a time during which food, sleep, and basic hygiene are neglected–are common among users of stimulant drugs and significantly affect dietary intake [69, 70]. Cocaine use is associated with alterations in food cues, eating disorders, and compulsive overeating [71]; all factors that impact on food security. For those who inject cocaine, elevated risks of infectious diseases, violence, victimization, overdose, mental health problems, poverty, incarceration, and sex trade involvement [72] have been found; all of these factors also interfere with stable access to food.

The findings that speed and mood disorders did not have significant interaction effects were surprising. Among regular amphetamine users, high rates of mental health problems such as psychosis have been found [73] and studies have shown bi-directional association. There may be a peripheral association between amphetamine use and food insecurity as some methamphetamine users lose their teeth from a condition known as meth mouth [74, 75]. This is a result of drug-induced psychological and physiological changes that lead to xerostomia, poor oral hygiene, frequent consumption of high-calorie, carbonated beverages, as well as teeth grinding and clenching [76] which can present barriers to healthy food intake.

### Study limitations

This study provided the opportunity to analyze a large representative population sample and present novel findings related to independent and synergistic associations between mood disorders and substance use in relation to food insecurity. However the findings are limited by the use of a cross-sectional design, insufficient details about substance use (e.g., dose, frequency, drug purity, routes of administration, period of life when substance used) and the measurement of household level food insecurity which cannot account for intra-household variations. Some households with food insecurity, which can include poor access to healthy foods even if they have access to enough food for energy and satiety needs, may have been screened out which could lead to underestimation of food insecurity. Although measuring for substances such as solvents, steroids and heroin and accounting for polysubstance use would have also been informative, the frequencies were too low in this sample to enable these types of analyses. Furthermore, caution must be used to avoid conflating being diagnosed with a mood disorder with having a mood disorder, as undiagnosed mental health problems may be very common. The results are specific to the province of British Columbia but may not be generalizeable to other settings (including populations in British Columbia not included in the sample). All data collected were self-reported and vulnerable to biases such as recall and social desirability. Finally, any other potentially important confounders could not be controlled for.

## Conclusions

The results of this study suggest that substance use and poor mental health are potential independent or interdependent factors that contribute to food insecurity. By tailoring prevention and intervention efforts, such as screening for the significant correlates reported in this study, strides may be made to alleviate the preventable public health issue of food insecurity. The results of some of the associations with food insecurity are difficult to interpret (e.g., potential potective effect of lifetime substance use related to food insecurity) and require further study. The conceptual framework (Fig 1) suggests that coping and resilience are underlying features that warrant further investigation as potential explanatory mechanisms that could mediate or moderate food insecurity, substance use, and mental ill health in the context of different macro-level drivers and mental health, nutrition, and behavioural pathways. Longitudinal research and further investigations of coping and resilience may discern explanatory mechanisms that could mediate or moderate substance use, poor mental health, and food insecurity. Finally, customized intervention research can help determine effective ways to reduce substance use, poor mental health, and food insecurity.
